# Non-cardiac comorbidities and mortality in patients with heart failure with reduced vs. preserved ejection fraction: a study using the Swedish Heart Failure Registry

**DOI:** 10.1007/s00392-019-01430-0

**Published:** 2019-02-20

**Authors:** Constantinos Ergatoudes, Maria Schaufelberger, Bert Andersson, Aldina Pivodic, Ulf Dahlström, Michael Fu

**Affiliations:** 1 Department of Molecular and Clinical Medicine, Institute of Medicine, Sahlgrenska Academy, University of Gothenburg, 416 85 Gothenburg, Sweden; 2 Department of Cardiology, Sahlgrenska Academy, University of Gothenburg, Gothenburg, Sweden; 3 Statistiska Konsultgruppen, Gothenburg, Sweden; 4 Department of Cardiology, Department of Medical and Health Sciences, Linköping University, Linköping, Sweden

**Keywords:** Heart failure, Comorbidities, Mortality, HFrEF, HFpEF

## Abstract

**Background:**

Heart failure (HF) and non-cardiac comorbidities often coexist and are known to have an adverse effect on outcome. However, the prevalence and prognostic impact of non-cardiac comorbidities in patients with HF with reduced ejection fraction (HFrEF) vs. those with preserved (HFpEF) remain inadequately studied.

**Methods and results:**

We used data from the Swedish Heart Failure Registry from 2000 to 2012. HFrEF was defined as EF < 50% and HFpEF as EF ≥ 50%. Of 31 344 patients available for analysis, 79.3% (*n* = 24 856) had HFrEF and 20.7% (*n* = 6 488) HFpEF. The outcome was all-cause mortality. We examined the association between ten non-cardiac comorbidities and mortality and its interaction with EF using adjusted hazard ratio (HR). Stroke, anemia, gout and cancer had a similar impact on mortality in both phenotypes, whereas diabetes (HR 1.57, 95% confidence interval [CI] [1.50–1.65] vs. HR 1.39 95% CI [1.27–1.51], *p* = 0.0002), renal failure (HR 1.65, 95% CI [1.57–1.73] vs. HR 1.44, 95% CI [1.32–1.57], *p* = 0.003) and liver disease (HR 2.13, 95% CI [1.83–2.47] vs. HR 1.42, 95% CI [1.09–1.85] *p* = 0.02) had a higher impact in the HFrEF patients. Moreover, pulmonary disease (HR 1.46, 95% CI [1.40–1.53] vs. HR 1.66 95% CI [1.54–1.80], *p* = 0.007) was more prominent in the HFpEF patients. Sleep apnea was not associated with worse prognosis in either group. No significant variation was found in the impact over the 12-year study period.

**Conclusions:**

Non-cardiac comorbidities contribute significantly but differently to mortality, both in HFrEF and HFpEF. No significant variation was found in the impact over the 12-year study period. These results emphasize the importance of including the management of comorbidities as a part of a standardized heart failure care in both HF phenotypes.

## Introduction

Heart failure (HF) is a serious progressive condition characterized by high mortality [1]. Because HF is often complicated with non-cardiac comorbidities that adversely affect prognosis [2, 3], targeting comorbidities has been increasingly advocated as being relevant to HF care. However, data about the relative prognostic impact of comorbidities, per se, or in combination, in HF with reduced ejection fraction (HFrEF) versus preserved EF (HFpEF) remain controversial. According to the European Society of Cardiology Heart Failure Pilot Survey, 74% of patients with HF had at least one comorbidity [4]. It is even common that patients with HF suffer from multiple comorbidities at the same time. Braunstein et al. [5] found that 40% of HF patients had ≥ 5 non-cardiac comorbidities (such as hypertension, diabetes, renal failure and anemia).

Several studies have reported different profiles in patients with HFrEF and HFpEF [6–9]. Higher average age and body mass index (BMI), more women, higher prevalence rates of atrial fibrillation and lower of ischemic heart disease (IHD) are common characteristics of the HFpEF group. The prevalence of non-cardiac comorbidities has previously been reported as higher in patients with HFpEF compared with patients with HFrEF, leading to the contention that these comorbidities may have a bigger impact on outcomes in the HFpEF population [10]. However, in a prospective observational cohort, our research group has reported similar non-cardiac comorbidities in patients with HFpEF compared with HFrEF patients [11]. Furthermore, the relative contribution of non-cardiac comorbidities to outcomes in HFpEF vs. HFrEF remains controversial [12–16]. Some of these studies examined only one comorbidity while others studied multiple non-cardiac comorbidities. Recently, Iorio et al. [17] demonstrated that non-cardiac comorbidities confer a similar contribution to outcomes in HFrEF and HFpEF patients in a community-based cohort. In contrast, Riedel et al. [18] reported that comorbidities had a higher contribution to mortality in HFpEF patients than in those with HFrEF.

In this study, we studied both prevalence and relative prognostic contributions of non-cardiac comorbidities to all-cause mortality in both HFrEF and HFpEF in a real-world HF population through access to the Swedish Heart Failure Registry (SwedeHF). Moreover, we examined whether an increasing number of non-cardiac comorbidities are associated with a higher risk of mortality and if this risk is similar in the two HF phenotypes.

The second purpose of the study was to examine possible variations of impact of each comorbidity on mortality over a 12-year study period. The treatment modalities of several comorbidities (e.g., hypertension, diabetes and stroke) have been improved over time [19–21]. However, whether these better treatments lead to a decreasing impact on mortality in patients with HF is unclear.

## Methods

The SwedeHF registry has been described previously [22]. In short, registration occurs either in hospitalized HF patients before discharge or at an outpatient visit. The single requirement for inclusion in the registry is clinician-judged HF. All data are collected and processed at the Uppsala Clinical Research Centre (UCR), Sweden. In the present analysis data from SwedeHF, the National Patient Register (NPR) and the Cause of Death Register were linked through the personal identity number, which is unique for all Swedish citizens for the collection of data on hospitalizations and causes of death. The protocol, registration form and annual reports are available at http://www.rikssvikt.se. Individual patient consent is not required but patients are informed of entry into the SwedeHF and allowed to opt out. The investigation conforms with the principles outlined in the “Declaration of Helsinki” [23].

### Study population

All patients registered in the SwedeHF between May 2000 and December 2012 constituted the study population. Exclusion criteria were (1) death during hospitalization, (2) incomplete information about EF and (3) existence of valve disease with clinical significance as it is reported in the SwedeHF. The population was followed for all-cause mortality, with the last follow-up in December 31, 2012. HFrEF was defined as EF < 50% and HFpEF as EF ≥ 50% [24].

### Non-cardiac comorbidities

The ten non-cardiac comorbidities included in the current investigation were hypertension, diabetes, stroke/transient ischemic attack (TIA), anemia, renal failure, pulmonary disease, liver disease, sleep apnea, gout and cancer within the past 3 years. In our study, hypertension was defined as reported hypertension, either in NPR or in SwedeHF, or systolic blood pressure ≥ 140 mmHg or diastolic pressure ≥ 90 mmHg at registration. Anemia was defined as reported anemia in NPR or hemoglobin (Hb) < 130 g/L for men and < 120 g/L for women at registration. Renal failure was defined as reported renal failure in NPR or an estimated glomerular filtration rate (e-GFR) of < 60, calculated according to the formula of the chronic kidney disease epidemiology collaboration (CKD-EPI), diabetes as reported either in NPR or SwedeHF and pulmonary disease as reported in either NPR or in SwedeHF or chronic obstructive pulmonary disease (COPD) in NPR. Remaining comorbidities (stroke/TIA, liver disease, sleep apnea and cancer within the past 3 years) were used as reported in the NPR.

### Statistical analysis

For categorical variables, *n* (%) is presented and for continuous variables mean with standard deviation (± SD). For comparison between groups, Fisher’s exact test was used for dichotomous variables, Mantel–Haenszel Chi-square test for ordered categorical variables and the Mann–Whitney *U* test for continuous variables. Age-adjusted analyses of patient characteristics were performed using logistic regression with HFrEF/HFpEF as the outcome variable, the variable of interest as the main effect variable and age as covariate.

The outcome is presented as event rate per 100 person-years with 95% confidence intervals (CIs), adjusted for age, and was obtained from Poisson regression. Adjusted incidence rate ratios (IRRs) for HFpEF vs. HFrEF with 95% CIs were also extracted from the same analysis. Median and interquartile range (IQR) were presented for the follow-up period.

Multivariable Cox regression analyses were performed for different variables at index date, calculating hazard ratios (HRs) with 95% CIs for mortality. The model was adjusted for age, sex, smoking, BMI ≥ 25 kg/m^2^, problematic alcohol use, IHD, dilated cardiomyopathy and atrial fibrillation or atrial flutter. The definition of IHD was a history of myocardial infarction or angina, coronary artery bypass grafting (CABG) or percutaneous coronary intervention (PCI), reported either in the NPR or in the SwedeHF. Problematic alcohol use was defined as reported either in the NPR or in the SwedeHF as current or previous problematic use. Atrial fibrillation or atrial flutter was defined as reported either in the NPR or in the SwedeHF. The missing values of smoking, BMI, IHD and dilated cardiomyopathy were treated as own categories in the adjustments. The impact of each comorbidity on mortality was examined separately for HFrEF and HFpEF, as well as the difference in the two impacts on all data together, including the group HFrEF/HFpEF and the interaction between this group and the comorbidity. To examine the trends over time in the contribution to mortality for each comorbidity, the similar models were performed separately for HFrEF and HFpEF, but also including the five consecutive periods (2000–2004, 2005–2006, 2007–2008, 2009–2010, 2011–2012) and the interaction between comorbidity and periods. The first period contained 4 years because of the small number of patients in the beginning of the registry. The assumption of proportional hazard was examined by including the interaction term between time in study and the comorbidity variable in each model and was found satisfactory.

All statistical analyses were performed using SAS statistical software version 9.4 (SAS Institute Inc., Cary, NC, USA). All tests were two-sided and *p* values < 0.05 were considered statistically significant.

## Results

**Fig. 1:**
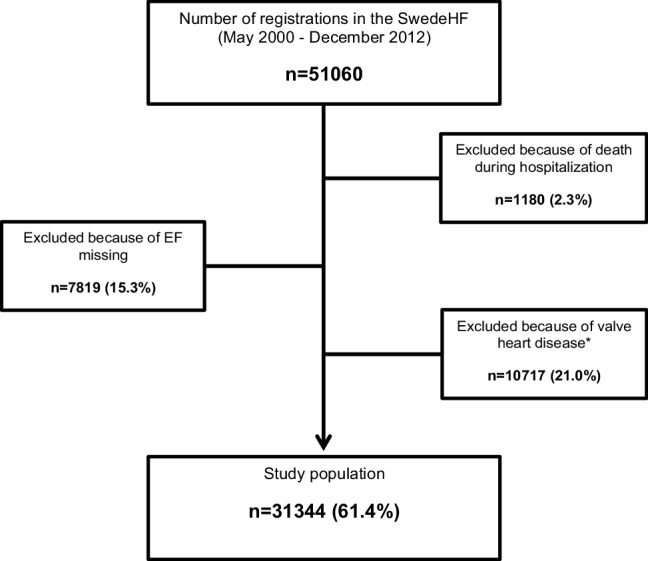
Study population: reasons for exclusion. *Valve disease with clinical significance as reported in the SwedeHF

The total number of registries in the SwedeHF from May 2000 through December 2012 was 51 060. After exclusion as per the criteria in “Methods”, the study population consisted of 31,344 individuals (Fig. 1). Of the 31,344 individuals, 24,856 (79%) had HFrEF and 6488 (21%) HFpEF. The median follow-up for the HFrEF group was 2.5 years (IQR 1.0–4.3) and for the HFpEF group 2.3 years (IQR 0.9–4.0).

The baseline characteristics of the participants are shown in Table 1. In the HFpEF group, there were significantly more women (53% vs. 31%) and patients were older, with a mean age of 77 vs, 71 compared with patients in the HFrEF group. Moreover, IHD was more common in the HFrEF group, whereas atrial fibrillation and flutter were more common in the HFpEF patients.

**Table 1:** Baseline characteristics of patients with HFrEF or HFpEF

Variable	Total (*n* = 31,344)	HFrEF (*n* = 24,856)	HFpEF (*n* = 6488)	*p* value	Age-adjusted *p* value
Age	72.5 (12.2)	71.4 (12.3)	76.8 (10.7)	< 0.0001	< 0.0001
Male sex	20,350 (64.9%)	17,286 (69.5%)	3064 (47.2%)		
Female sex	10,994 (35.1%)	7570 (30.5%)	3424 (52.8%)	< 0.0001	< 0.0001
BMI ≥25 kg/m^2^	8888 (61.8%)	6998 (60.6%)	1890 (66.3%)	< 0.0001	< 0.0001
BMI ≥ 30 kg/m^2^	3704 (25.7%)	2786 (24.1%)	918 (32.2%)	< 0.0001	< 0.0001
Smoking					
Never	10,177 (41.1%)	7905 (39.5%)	2272 (47.7%)		
Previous	10,972 (44.3%)	9000 (45.0%)	1972 (41.4%)		
Current	3606 (14.6%)	3087 (15.4%)	519 (10.9%)	< 0.0001	< 0.0001
Previous or current alcohol problems	2379 (7.6%)	2005 (8.1%)	374 (5.8%)	< 0.0001	0.53
IHD	17,778 (57.4%)	14,437 (58.9%)	3341 (52.0%)	< 0.0001	< 0.0001
Dilated cardiomyopathy	3909 (12.9%)	3732 (15.5%)	177 (2.8%)	< 0.0001	< 0.0001
Atrial fibrillation/flutter	16,260 (51.9%)	12,328 (49.6%)	3932 (60.6%)	< 0.0001	< 0.0001
Non-cardiac comorbidities					
Hypertension	21,684 (69.2%)	16,418 (66.1%)	5266 (81.2%)	< 0.0001	< 0.0001
Diabetes	8732 (27.9%)	6780 (27.3%)	1952 (30.1%)	< 0.0001	< 0.0001
Stroke/TIA	5041 (16.1%)	3778 (15.2%)	1263 (19.5%)	< 0.0001	0.0003
Anemia	11,231 (35.8%)	8399 (33.8%)	2832 (43.7%)	< 0.0001	< 0.0001
Renal failure	14,706 (47.1%)	11,155 (45.0%)	3551 (54.9%)	< 0.0001	0.47
Lung disease	8954 (28.6%)	6676 (26.9%)	2278 (35.1%)	< 0.0001	< 0.0001
Liver disease	501 (1.6%)	370 (1.5%)	131 (2.0%)	0.0037	< 0.0001
Sleep apnea	1132 (3.6%)	835 (3.4%)	297 (4.6%)	< 0.0001	< 0.0001
Gout	1329 (4.2%)	998 (4.0%)	331 (5.1%)	0.0002	0.026
Cancer within last 3 years	4108 (13.1%)	3094 (12.4%)	1014 (15.6%)	< 0.0001	0.0042

For categorical variables, *n* (%) is presented. For continuous variables, mean (SD) is presented

*HFrEF* heart failure with reduced ejection fraction, *HFpEF* heart failure with preserved ejection fraction, *BMI* body mass index, *IHD* ischemic heart disease, *TIA* transient ischemic attack

### Prevalence of non-cardiac comorbidities

**Fig. 2:**
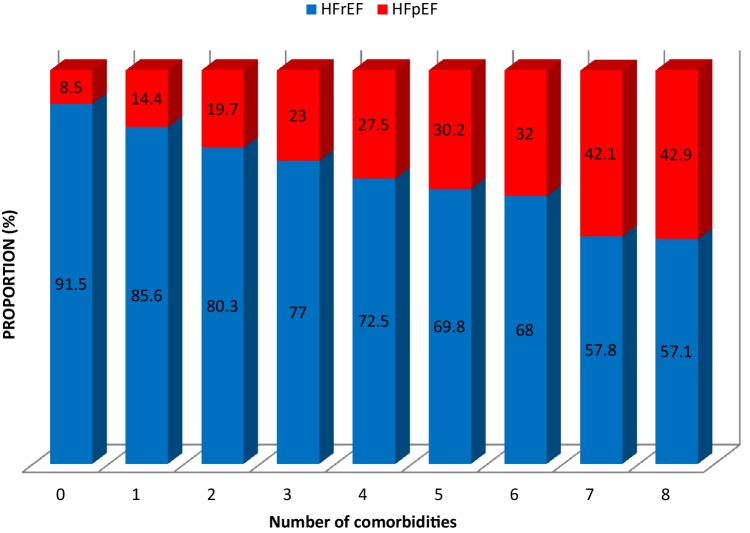
Distribution of the HF population (HFrEF vs. HFpEF) stratified by number of comorbidities

Patients with HFpEF had a higher prevalence of hypertension, diabetes, stroke/TIA, anemia, pulmonary disease, liver disease, sleep apnea, gout and cancer. There was no difference in the prevalence of renal failure between groups after age adjustment. The number of non-cardiac comorbidities per patient was higher in the HFpEF group (mean 2.9 ± 1.5) compared with the HFrEF group (mean 2.4 ± 1.5). The highest number of comorbidities per patient was eight in both groups. Figure 2 depicts the distribution of the study population (HFrEF vs. HFpEF) stratified by the number of comorbidities. The HFpEF patients consisted only of 8% and 14% of the groups with none and one comorbidity, respectively, compared with 42% and 43% in the group with seven and eight comorbidities, respectively.

### Contribution of non-cardiac comorbidities to all-cause morality

Death occurred in 8529 (34.3%) patients in the HFrEF group with an age-adjusted event rate per 100 person-years of 11.1 (CI 95% 10.9–11.4) and in 2614 (40.3%) patients in the HFpEF group with an age-adjusted event rate per 100 person-years of 10.6 (CI 95%CI 10.1–11.0), which had a slightly lower mortality rate: IRR 0.95 (95% CI 0.91–0.99), *p* = 0.018.

**Fig. 3:**
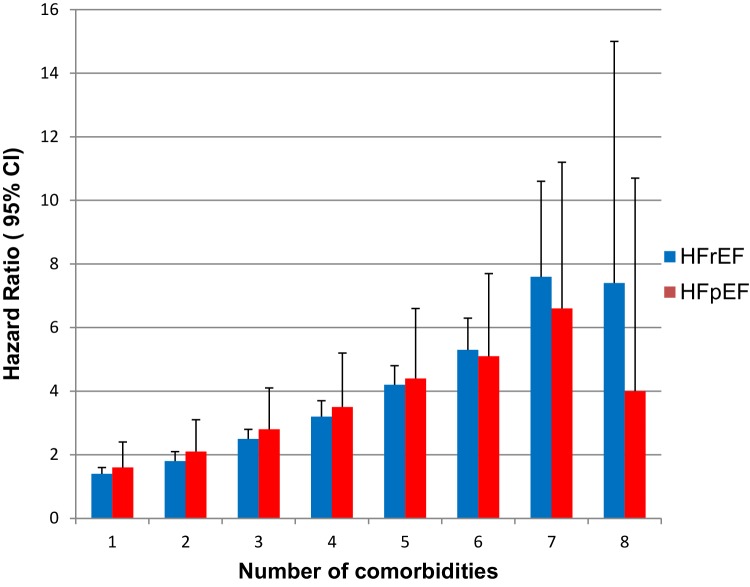
The adjusted effect of number of comorbidities on mortality for HFrEF and HFpEF

An increased number of comorbidities was associated with higher risk of mortality in both HF phenotypes, according to the multivariable survival analysis (Fig. 3). The adjusted Cox proportional hazard model for the prediction of time to death by non-cardiac comorbidity for both HFrEF and HFpEF patients, as well as the interaction with EF is presented in Table 2. Diabetes, stroke/TIA, anemia, renal failure, pulmonary disease, liver disease, gout and cancer were all independent predictors of mortality in both groups. Hypertension was associated with a reduced risk of mortality in the HFpEF group, as in the HFrEF group, with a border level of statistical significance. Sleep apnea was not associated with mortality in either group. The interaction analysis showed that diabetes (HR 1.57, 95% confidence interval [CI] [1.50–1.65] vs. HR 1.39, 95% CI [1.27–1.51], *p* = 0.0002), renal failure (HR 1.65, 95% CI [1.57–1.73] vs. HR 1.44, 95% CI [1.32–1.57], *p* = 0.003) and liver disease (HR 2.13, 95% CI [1.83–2.47] vs. HR 1.42, 95% CI [1.09–1.85] *p* = 0.02), HFrEF vs. HFpEF, respectively, had a higher impact in HFrEF patients, whereas pulmonary disease contributed to a higher risk in patients with HFpEF (HR 1.66, 95% CI [1.54–1.80] vs. HR 1.46, 95% CI [1.40–1.53], *p* = 0.007), HFpEF vs. HFrEF, respectively. No statistically significant interaction was found for stroke/TIA, anemia gout or cancer within the past 3 years.

**Table 2:** Adjusted Cox proportional hazard models for prediction of time to death by non-cardiovascular comorbidities per ejection fraction category and interaction with ejection fraction

Variable	HFrEF		HFpEF		*p* value Interaction with EF
	Hazard ratio (95%CI)	*p* value	Hazard ratio (95%CI)	*p* value	
Hypertension	0.96 (0.91–1.00)	0.051	0.85 (0.77–0.93)	0.0007	0.0063
Diabetes	1.57 (1.50–1.65)	< 0.0001	1.39 (1.27–1.51)	< 0.0001	0.0002
Stroke/TIA	1.36 (1.29–1.43)	< 0.0001	1.30 (1.19–1.43)	< 0.0001	0.10
Anemia	1.70 (1.63–1.78)	< 0.0001	1.65 (1.53–1.79)	< 0.0001	0.42
Renal failure	1.65 (1.57–1.73)	< 0.0001	1.44 (1.32–1.57)	< 0.0001	0.0031
Lung disease	1.46 (1.40–1.53)	< 0.0001	1.66 (1.54–1.80)	< 0.0001	0.0066
Liver disease	2.13 (1.83–2.47)	< 0.0001	1.42 (1.09–1.85)	0.0084	0.015
Sleep apnea	1.11 (0.96–1.27)	0.15	1.17 (0.94–1.45)	0.16	0.83
Gout	1.57 (1.43–1.72)	< 0.0001	1.38 (1.17–1.62)	0.0001	0.051
Cancer	1.35 (1.28–1.43)	< 0.0001	1.35 (1.22–1.49)	< 0.0001	0.84

Adjustment for age, sex, smoking, BMI ≥ 25 kg/m^2^, problematic alcohol use, IHD, dilated cardiomyopathy and atrial fibrillation or atrial flutter

*HFrEF* heart failure with reduced ejection fraction, *HFpEF* heart failure with preserved ejection fraction, *TIA* transient ischemic attack

### Trend of contribution of non-cardiac comorbidities to all-cause morality in the past decade

**Fig. 4:**
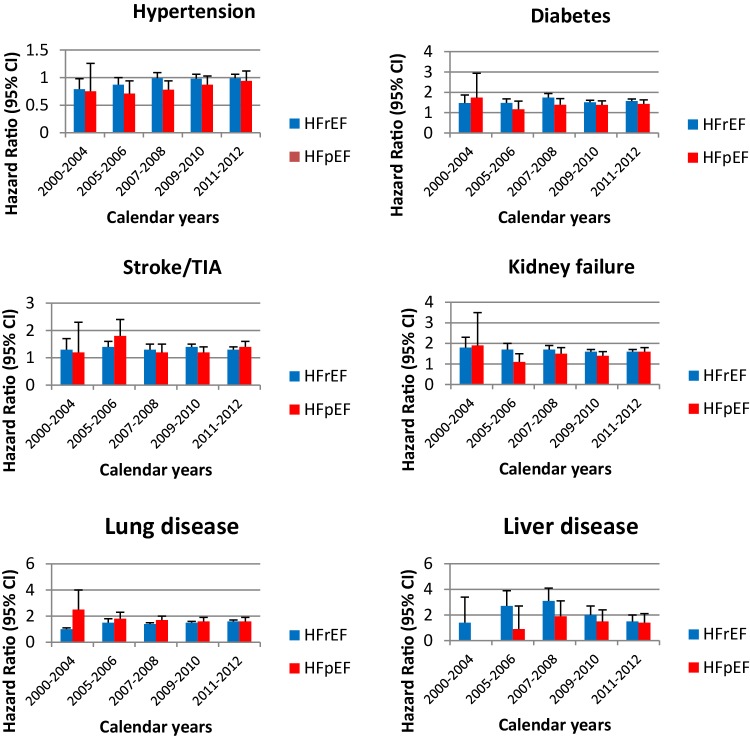
The adjusted effect of non-cardiac comorbidities for HFrEF and HFpEF on mortality (trend over time)

During the follow-up (2000–2012), no statistically significant interaction between periods and the effect of each comorbidity on mortality was found, except for pulmonary and liver disease, which had lower risk in the HFrEF group during the first period (2000–2004) (Fig. 4).The low rate of registration in SwedeHF during this period resulted in wider HR confidence intervals compared to the other time periods.

## Discussion

Using data from the SwedeHF registry linked to Swedish National Patient Register and the Cause of Death Register, our findings contribute important information about the contribution of non-cardiac comorbidities to mortality in both HFrEF and HFpEF patients. To our knowledge, this is probably the largest analysis in an unselected HF population on this subject.

In view of the existing data about prevalence and impact of non-cardiac comorbidities in HFpEF and HFrEF [12–14, 16] our results are clinically relevant because they extend knowledge into three areas: (1) both HFrEF and HFpEF patients have a high burden of non-cardiac comorbidities, (2) an increased number of comorbidities increased the likelihood of HFpEF and (3) regardless if HFpEF or HFrEF, an increased number of non-cardiac comorbidities was associated with increased mortality. Our results are mainly in line with a recent study by Iorio et al. [17]. However, in our study, we investigated the impact of up to eight comorbidities compared with Iorio et al. [17] who studied ≥ 3. The lower risk in patients with eight comorbidities compared with those with seven, as shown in Fig. 3, probably depends on the relatively low number of patients and outcomes in that group, which explains the wide confidence interval. Despite that non-cardiac comorbidities were more common in HFpEF in general, renal failure had the same prevalence in HFpEF and HFrEF patients after age adjustment. In contrast, the prevalence of renal failure varied between previous studies [12, 16, 17]. We believe that our data are robust because of the large sample size and that e-GFR for the estimation of kidney function was applied.

Our data suggest a notable contribution of non-cardiac comorbidities to mortality in both HF phenotypes. However, our results differ from previous studies showing a similar risk between the two HF groups [14, 16, 17]. There might be several explanations for the discrepancy, the first of which concerns the use of different definitions of preserved EF [13, 14]. Another pertains to how possible confounders were adjusted in the multivariable models in different studies [17]. In our study, a significant number of variables that may act as confounders were included in the analysis.

In our study, pulmonary disease had a higher hazard of mortality in HFpEF patients. In the SwedeHF, COPD is not a stand-alone variable, but rather a part of pulmonary disease, which might explain why in previous studies COPD was found to have a similar hazard in both HFpEF and HFrEF [17]. However our results regarding pulmonary disease are similar to the results from Ather et al. regarding COPD [12]. We found that diabetes, renal failure and liver disease had a greater contribution to mortality in patients with HFrEF than in patients with HFpEF. Hypertension seems to be a protective factor for mortality in both HF groups, with a slightly higher impact in HFpEF. A possible explanation to the protective nature of hypertension is that it reflects the beneficial effect of hypertensive treatment on mortality. However, we do not have data on blood pressure control over time, which is a limitation of the study given that it has recently been shown that a u-shaped relationship between blood pressure and mortality might exist in HF populations [25]. Sleep apnea was not associated with higher mortality in either group. The prevalence of sleep apnea in our study was lower than the prevalence reported in earlier studies [26]. It is, therefore, reasonable to assume that it might be under-diagnosed. The low prevalence might explain why sleep apnea was not associated with higher mortality.

On the basis of our findings, a greater focus on the recognition and treatment of comorbidities in both HF categories is justified. This approach may be particularly relevant for HFpEF where no therapies are available to reduce mortality, but also for HFrEF where the burden of non-cardiac comorbidities was also high. Polypharmacy is common in patients with HF that becomes more complicated when medical treatment for one or more comorbidities is indicated. A multidisciplinary management may be beneficial for these patients.

We have not seen any variations on the impact of mortality for any of the comorbidities examined and certainly no decreasing effect over time in the past decade in neither HFrEF nor HFpEF patients despite that treatment for some of these comorbidities has improved. There are several possible explanations: (1) the investigated period might be too short for changes in the treatment of examined diseases to affect prognosis of the disease itself and, therefore, the impact on mortality in HF patients. (2) The improved therapy of non-cardiac comorbidities is not sufficient to improve outcome in HF and (3) management of non-cardiac comorbidities remains suboptimal in many patients with HF. In addition, it is possible that many of the non-cardiac comorbidities remain under-diagnosed and, therefore, continuously negatively affect prognosis or that many of the non-cardiac comorbidities are diagnosed too late so that they cannot effectively respond to therapy.

## Strengths and limitations

The strengths of this study are the large sample size in a real-world cohort and that the data were linked to the Swedish national health data registries, which are obligatory for all health providers in Sweden. In addition, we were able to study the prevalence and prognostic impact of non-cardiac comorbidities during a 12-year period that encompasses many significant achievements not only in HF but also in the management of non-cardiac comorbidities.

Our study has some limitations. First, our observational study is subject to confounding and selection bias. Indeed, although we performed extensive adjustments, we cannot rule out potential residual confounding. Second, there was limited information on comorbidities regarding the degree of severity or staging of the disease, or disease duration, which would allow a more detailed investigation on the contribution to mortality for each comorbidity. In this study, patients with significant valve disease were excluded from the analysis. The only variable available in SwedeHF is the presence of valve disease of clinical significance. Since data on the grade or the type of valve disease were not available, these patients were excluded from the analysis.

## Conclusions

In a large HF cohort that included both HFrEF and HFpEF, non-cardiac comorbidities had a significant contribution to mortality in both phenotypes with some notable intergroup differences. These findings might imply a need for high-priority optimal management of non-cardiac comorbidities in both HF phenotypes, particularly in HFpEF, as other effective therapies are still lacking but also in HFrEF where the burden of non-cardiac comorbidities is high as well. The prognostic impact of the comorbidities examined in this analysis remained unchanged over the 12-year study period. Possible explanations, without excluding the possibility that the study period was too short for changes in the treatment to influence the impact, could be the suboptimal management of these comorbidities, or that the improved therapy of non-cardiac comorbidities was not sufficient to improve outcome in HF.
